# A cross-sectional study on the microbiological quality and safety of raw chicken meats sold in Nairobi, Kenya

**DOI:** 10.1186/1756-0500-7-627

**Published:** 2014-09-10

**Authors:** Joyce Arua Odwar, Gideon Kikuvi, James Ngumo Kariuki, Samuel Kariuki

**Affiliations:** Institute of Tropical Medicine and Infectious Diseases, Jomo Kenyatta University of Agriculture and Technology, P.O Box 62000–00200, Nairobi, Kenya; Centre for Public Health Research, Kenya Medical Research Institute, P.O Box 20752–00202, Nairobi, Kenya; Centre for Microbiology Research, Kenya Medical Research Institute, P.O Box 43640–00100, Nairobi, Kenya

**Keywords:** Raw retail chicken meat, E. coli/coliforms, Microbial counts

## Abstract

**Background:**

Chicken is a rich source of meat protein and is increasingly being consumed in urban areas in Kenya. However, under poor hygienic environment, raw chicken meat presents an ideal substrate supporting the growth of pathogenic *Escherichia coli* and Coliform bacteria indicating the potential presence of other pathogenic bacteria; this may constitute a major source of food-borne illnesses in humans. This study sought to assess the microbiological quality and safety of raw chicken meat sold in Nairobi, Kenya by determining the *E. coli*/coliform contamination levels as well as the antimicrobial resistance patterns and pathogenicity of *E. coli* isolated.

**Findings:**

We conducted a Cross-sectional study to collect two hundred raw chicken samples that were randomly purchased between the periods of August 2011-February 2012. Enumeration of bacteria was done using 3 M Petri film *E. coli*/Coliform count plates, isolation and identification of *E. coli* through standard cultural and biochemical testing, antimicrobial susceptibilities interpreted according to criteria set by the Clinical and Laboratory Standards Institute (2012) while Polymerase chain reaction assays were used to determine presence of virulence genes in isolated *E. coli*. Data was analyzed using SPSS version 17.0. Contamination rates were 97% and 78% respectively for Coliform bacteria and *E. coli*. Seventy six percent of samples fell under the unacceptable microbial count limit (>100 cfu/ml) and significant differences in the *E. coli*/coliform counts (p < 0.001) were observed among the chicken retail outlets with samples from supermarkets having the lowest level of contamination compared to the rest of the retail outlets. Seventy five percent of the isolates were resistant to at least one of the 12 antibiotics tested with resistance to tetracycline being the highest at 60.3%. In addition 40.4% *E. coli* isolates were positive for the ten virulence genes tested.

**Conclusion:**

Raw retail chicken meats in Nairobi are not only highly contaminated, but also with potentially pathogenic and multi-drug resistant strains of *E. coli*. It will be important for public health authorities and retail chicken processing outlets to collaborate in ensuring adherence to set out principles of hygienic processing and handling of chicken meats in order to reduce potential risks of infection.

## Findings

### Background

Ensuring safe food supply has been one of the major challenges and concerns for producers, consumers and public health officials in both developing and developed countries. This is because foods excessively contaminated with pathogenic and spoilage micro-organism are undesirable and can cause food borne illnesses [1–3]. Such illnesses cost billions of dollars in medical care and sometimes even result to death [4]. Several epidemiological reports have implicated foods from animal origin as major vehicles associated with illnesses caused by food borne pathogens [5–7]. Coliform bacteria, especially fecal coliforms, are good microbial indicators of the potential presence of disease causing bacteria and also show the general sanitary quality of the food. Food contamination by *Escherichia coli* is closely associated with fecal contamination. This is because *E. coli* are the most prevalent commensal enteric bacteria in animals and humans and are also important zoonotic agents that can be implicated in animal and human infectious diseases [8].

Raw or undercooked chicken meat is particularly prone to contamination. The microbiological quality of chicken meat as purchased by consumers depends mostly on; the slaughter process, sanitation during processing and packaging, maintenance of adequate cold chain storage from the processing to the retail level and to the consumer and finally sanitation during handling at the retail end [9–12]. Chicken meat can also act as a reservoir of drug resistant bacteria. Antimicrobial resistance among *E. coli* in food animals such as chicken is of increasing concern due to the potential for transfer of these resistant pathogens to the human population [13–15].

In urban areas such as Nairobi, marketing of chicken products is generally undertaken in retail outlets such as supermarkets, local butcheries located in different geo-socio-economic status and even from street vendors in some low income settings. Public health research in countries such as the United States of America focusing on food qualities demonstrated that stores in low socio-economic status populations, because of a higher prevalence of food safety violations, were shown to be consistently exposed to food that is of lower microbial quality. Because of this access pattern, such populations are placed at increased risk of food-borne illnesses [16, 17]. In Kenya, there is a paucity of data on coliform contamination and antimicrobial resistant *E. coli* in raw retail chicken supplied to retail outlets in the city. Studies related to antimicrobial resistant *E. coli* have been done on isolates from farm animals and chicken carcass samples from slaughter [18] but not from raw retail chicken which is made available to consumers. Furthermore, no studies are available on the contamination levels in chicken meat available to populations living in different socio-economic status yet, these data are essential for performing risk assessment and risk management for food safety. This study reports on the microbiological quality and safety of chicken meat available to populations who purchase from different retail outlets (supermarkets, retail outlets in high income areas, retail outlets in middle income areas and retail outlets in low income areas) in the city of Nairobi.

## Methods

### Study area and sampling

The study was conducted in Nairobi County. Nairobi is the administrative and commercial capital in Kenya and home to thousands of businesses including the retail chicken business.. There is a huge disparity in income levels and population densities in Nairobi. The people living in the western suburbs are generally the more affluent while the lower and middle-income populations dominate the eastern suburbs [19].

In a cross-sectional study, chicken samples were randomly purchased from August 2011 to February 2012 from different retail outlets spread over 28 locations in Nairobi. In order to take into account compounding factors of socio-economic status within Nairobi, the retail outlets where samples were purchased were classified into supermarkets, shops from high end areas (low densely populated, up-market residential suburbs), shops from middle end areas (middle densely populated areas further classified into high middle and low middle income areas) and shops from low end areas (densely populated slums and informal settings). Classification of locations into these groups was done based on a study on residential segregation in Nairobi [20]. A total of two hundred chicken samples (n = 39, supermarkets; n = 39 high end area retail outlets; n = 84, high middle end area retail outlets; n = 20, low middle end area retail outlets and n = 18, low end area retail outlets) were purchased. Both freshly slaughtered as well as samples that have been in storage in freezers were included in the study. Packaged chicken samples were transported in cool boxes to the laboratory for bacterial isolation within 1 hour from time of collection. Samples were processed at the Kenya Medical Research Institute, Centre for Microbiology Research laboratories, Kenyatta hospital compound. Ethical approval to perform this study was obtained from the Ethics Review Committee at Kenya Medical Research Institute, Reference SSC No. 2036.

### Bacteriological analysis

Enumeration of *E. coli* and coliform bacteria from the chicken samples was performed as described in the Association of Analytical Communities International, official methods of analysis using 3 M petrifilm *E. coli*/Coliform count plates [21] with slight modification. The samples, which included the skin of the chicken along with the meat itself, were aseptically removed from the package and 100 gram piece of chicken was weighed and placed in 100 ml sterile distilled water. One ml of the rinse water from the sample was placed onto the center of the bottom film and covered carefully avoiding entrapment of air bubbles and the plates from all samples incubated for 24 ± 2 hrs at 35°C. Colony counts for each petrifilm were done the following day and the microbial count results converted to the base 10 logarithm of the number of colony forming units per ml (cfu/ml) rinse water obtained from the samples. Typical *E. coli* colonies growing on the petrifilm were then selected and sub-cultured in MacConkey agar to obtain pure colonies. After a series of biochemical tests for confirmative identification [22], positive *E. coli* isolates were stored at -80°C in trypticase soy broth for further antimicrobial susceptibility tests and PCR to determine the presence of virulence genes.

### Antimicrobial susceptibility testing

Using the Kirby-Bauer disc diffusion method [23], *E. coli* were tested for their susceptibility to 12 commonly used antimicrobials on disks containing; Ampicillin (AMP 10 μg), Amoxycillin-clavulanic acid (AMC, 30 μg), Tetracycline (TE 30 μg), sulphamethoxazole-trimethoprin (SXT 30 μg), Ciprofloxacin (CIP 5 μg), Ceftriaxone (CRO 30 μg), Ceftazidime (CAZ 30 μg), Kanamycin (K 30 μg), Streptomycin (S 10 μg), Gentamicin (CN 10 μg), Nalidixic acid (NA 30 μg) and Chloramphenicol (C 30 μg). The concentrations of the antimicrobial disks were selected based on the internationally recognized standards and guidelines on antimicrobial routine testing and reporting on enterobacteriaceae provided by the Clinical and Laboratory Standard Institute. *E. coli* strain 25922 was used to control for bacterial growth and potency of antibiotic disks. Inoculated agar plates were incubated at 37°C for 24 h. The susceptibility zones were also measured and interpreted according to criteria set by the Clinical and Laboratory Standards Institute [24].

### Bacterial DNA extraction and detection of virulence genes in *E. coli*

DNA from the isolated *E. coli* as well as from 5 control strains was extracted following the procedures described by Ehrt and Schnappinger [25], with slight modifications. A loop full of overnight bacterial culture was suspended in 1 ml of sterile distilled water and then boiled for 10 minutes at 95°C. The cell mixture was centrifuged for 5 minutes at 14,000 rpm and supernatant was used as the DNA template for PCR amplification. Ten PCR primers were used to detect the target genes enumerating toxins in pathogenic *E. coli* [26]. A multiplex PCR system was optimized by the progressive incorporation of primers corresponding to the different genes and several combinations of melting temperatures, primer concentrations and DNA template concentration. PuReTaq ready to go PCR beads (GE Health care) were used for PCR. The total reaction volume in each PCR tube was 25 μl containing 3 μl template DNA, 2 μl primer (0.2 μl of each primer) and 20 μ sterile PCR water. PCR was done under the following conditions; initial denaturation at 94°C for 5 minutes. This was followed by 35 cycles of denaturation at 94°C for 1 minute, annealing at 56°C for 30 seconds and extension at 72°C for 1 minute. The final step was amplification at 72°C for 10 minutes. Amplicons were then subjected to agarose gel electrophoresis and viewed under UV light.

### Statistical analysis

Statistical analysis including means, medians and standard deviations, was conducted by means of SPSS version 17.0 software. To determine differences in microbial counts in chicken samples purchased from among the various retail outlets, ANOVA was used after converting the counts to base 10 logarithms. The chi-square test was used to assess any statistical significant association between antimicrobial resistances in the *E. coli* isolated with regard to retail outlet classification from where the raw chicken was purchased.

## Results

### Contamination rate and microbial count in raw retail chicken

**Figure 1:**
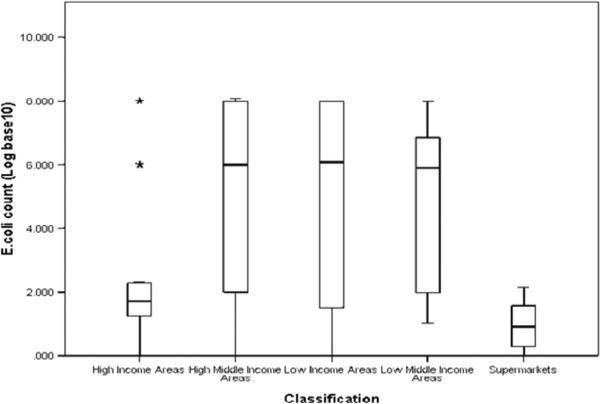
**Distribution of** ***E. coli*** **count among the 5 retail outlet classifications.** Counts were evenly distributed., the size of the boxes for high- middle income, low-middle income and low income areas indicate that the middle 50% *E. coli* counts are spread out for these groups while for high class butcheries and supermarkets the box sizes indicate that the middle 50% of the counts are clumped together.

**Figure 2:**
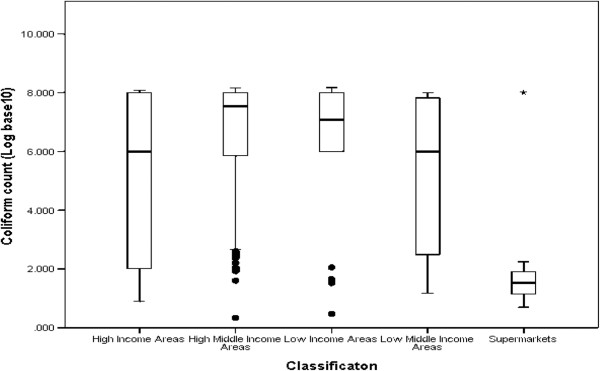
**Distribution of Coliform count among the 5 retail outlet classifications.** Apart from supermarkets, the other 4 retail outlet classifications have bulk of the count concentrated on the high end of the scale.

Contamination rate of chicken samples by total coliform bacteria was found to be 97% while contamination by *E. coli* was 78%. The average *E. coli* and coliform counts for all samples in general were observed to be above the acceptable range for *E. coli* counts as set by the Hazard Analysis and Critical Control Point system (HACCP), developed by Food and Agriculture Organization (FAO) and adopted by the Codex Alimentarius Commission (3.911 and 5.0261Log_10_cfu/ml respectively). According to this system, the acceptable food safety range is 100 cfu/ml or less (2.000 Log_10_cfu/ml or less), the marginal or intermediate range is over 100 cfu/ml but not above 1000 cfu/ml (over 2.000 but not above 3.000 Log_10_cfu/ml) and the unacceptable range is above 1000 cfu/ml (above 3.000 Log_10_cfu/ml) [27]. Only 80 (40%) out of a total 200 retail chicken samples that were purchased fell under the acceptable range for *E. coli* counts. 76% of samples fell under the unacceptable range for total coliforms. As shown in Tables 1 and 2 supermarkets had the highest percentage of samples within acceptable food safety ranges while average counts of samples from the rest of the retail outlets were within marginal or unacceptable counts of above 2.000 Log_10_cfu/ml. There was a statistical significant difference in the *E. coli* count (F (4,175) =16.676; MSE = 106.576; p < 0.001) and coliform counts (F (4,179) =18.37; MSE = 105.097; p < 0.001) among the outlets with supermarkets having the lowest *E. coli* and coliform count compared to the rest. Graphical evidence of the differences of *E. coli* and coliform counts observed in the different retail outlet classifications are also shown in Figures 1 and 2.

**Table 1:** **Descriptive statistics of microbiological count of raw chicken meats from 5 different classifications of retail outlets**

		Total count log _10_ CFU/ml of carcass rinse				
Bacteria	Summary statistics	Supermarkets	H.I.A butcheries	H.M.I butcheries	L.M.I butcheries	L.I.A butcheries
*E.coli*	Average log_10_	0.9376	2.6510	5.0007	4.8084	4.9882
	Median	0.9031	1.7243	6.0000	5.9031	6.0792
	SD	0.6794	2.3638	2.8155	2.5910	3.1096
	Minimum	0.0000	0.0000	0.0000	1.0410	0.0000
	Maximum	2.1460	8.0000	8.0790	8.0000	8.0000
Coliforms	Average log_10_	1.7239	5.0769	6.1855	5.2953	6.0111
	Median	1.5315	6.0000	7.5374	6.0000	7.0731
	SD	1.3324	2.9304	2.3052	2.5475	2.6770
	Minimum	0.6990	0.9030	0.3420	1.1760	0.4770
	Maximum	8.0000	8.0790	8.1610	8.0000	8.1760

**H.I.A butcheries-**High income area butcheries, **H.M.I butcheries-**High-middle income area butcheries.

**L.M.I-** Low-middle income area butcheries, **L.I.A-** Low income area butcheries.

**Table 2:** **Assessment of Microbial count results of raw chicken meat from the different retail markets**

Assessment of microbial counts Log _10_cfu/ml						
		% acceptable samples		% marginal samples		% unacceptable samples
Retail outlet	***E.coli***	Coliforms	***E.coli***	Coliforms	***E.coli***	Coliforms
**Supermarket**	97%	84%	3%	13%	0%	3%
**H.I.A butcheries**	69%	26%	10%	15%	21%	59%
**H.M.I butcheries**	29%	7%	12%	14%	60%	79%
**L.M.I butcheries**	25%	10%	10%	20%	65%	70%
**L.I.A butcheries**	16%	33%	6%	6%	78%	61%

### Prevalence of antimicrobial resistant *E. coli*isolated from raw retail chicken

Seventy five percent (117 out 156) of the *E. coli* isolated exhibited resistance to at least one of the 12 antibiotics tested. As seen in Table 3, prevalence of antimicrobial resistance was highest for tetracycline followed by sulphamethoxazole-trimethoprin, ampicillin and streptomycin. Only 24.5% of the isolates were fully sensitive to all antibiotics while 42.9% were resistant to 3 or more antibiotics especially to the above mentioned antimicrobials. There was no significant difference in the prevalence of antimicrobial resistance among the 5 classifications of retail outlets (χ^2^n-1 = 4.178; d.f = 4; p = 0.382). However, samples from supermarkets had the highest prevalence (84.6%) while samples from outlets in low income areas had the lowest prevalence of antimicrobial resistance (62.5%).

**Table 3:** **Antimicrobial resistance profiles among** ***E. coli*** **isolated in retail chicken meat (n = 156)**

***Antibiotic***	Frequency of resistant isolates	%
**AMC**	4	2.6
**SXT**	77	49.4
**TE**	94	60.3
**AMP**	53	34
**CIP**	7	4.5
**CAZ**	0	0
**K**	8	5.1
**S**	47	30.1
**CRO**	18	11.5
**CN**	1	0.6
**NA**	23	14.7
**C**	21	13.5

### Prevalence of *E. coli*isolates harboring virulence markers

Of the *E. coli* isolated from raw retail chicken meat, 40.4% carried at least one of 10 virulence genes tested and specific for 5 known diarrheagenic *E. coli*. Of the *E. coli* positive for virulence genes in this study, ETEC was the most common pathogenic *E. coli* (61%) while the least common was EAggEC (5%) (Table 4).

**Table 4:** **Classification of pathogenic** ***E. coli*** **identified from retail chicken meat**

Classification of pathogenic ***E. coli***	Frequency of isolates	%
EPEC	13	20.6
ETEC	38	60.3
EIEC	4	6.3
EaggEC	3	4.8
STEC	5	7.9

## Conclusion

The *E. coli* contamination rate observed in this study was almost similar to that observed in studies conducted in other developing countries. For instance, contamination rates of 98% were observed in the north east part of India, 100% in Cameroon and 100% in Vietnam [28–30]. Similar high recovery rates have been observed in studies from developed countries with contamination rates as high as 100% in Japan, 89% Minnesota, U.S.A, and 90% in two states in Australia [31–34]. Similarly, studies in Hanoi, Vietnam, Morocco and some states in America have shown that the percentage of retail chicken samples which fall under the unacceptable food safety range were also high and that perishable produce items, including poultry, available in markets in low-socio economic status census tracts had higher microbial indicator counts compared to markets in high-socio economic status due to a higher prevalence of food safety violations [30, 35–37, 16, 17]. The results observed in this study might be due to factors common in Nairobi such as home slaughter of chicken by small scale poultry producers instead of slaughter at private or municipal government approved chicken slaughter houses thus increasing the potential risks for contamination due to bio-security flaws [13]. All samples from supermarkets and a majority of samples from high income butcheries were products of government approved private chicken slaughter houses. Majority of carcasses in Nairobi tend to be lumped together in one large container or sack as they are being transported in ambient temperature thus exposing them to the open air as well as allowing transfer of contaminants from one carcass to another and subsequent microbial multiplication. Studies in Iran and Switzerland showed that there were significant differences in contamination rates between individually packed and unpacked chicken meat samples [38, 39]. Use of freezers and cooling temperatures for storage and display in some outlets from high middle income and low income areas could have resulted to few samples falling under the acceptable food safety range in these outlets as seen from the outliers in Figure 1 [40, 41]. In most retail outlets, the degree of physical contact between different kinds of meat on display such as beef, fish and mutton may have led to cross contamination of different meats. Also, use of bare hands in handling meat, utensils and money at the same time as observed may have increased chances of microbial contamination [42]. In contrast to the findings in this study, a similar recent study in Accra, Ghana found that even though hygienic conditions in supermarkets were generally better than those in local markets and farms, there was no significant difference between the microbial counts for these retail outlets and they all had low microbial counts [43]. The authors attributed this observation to the deliberate efforts by the food sector in improving the hygienic procedures in the processing of poultry over the years.

Similar prevalence of antibiotic resistance in *E. coli* isolated from retail chicken have been observed in other studies ranging from 40.6% in Japan, 52% in Iceland, 84.6% in Minneapolis, U.S.A, 83.8% in Vietnam and even 100% isolates in Senegal being resistant to one or more antibiotics [31, 44–47]. The high prevalence of resistance in poultry meat isolates is alarming given the evidence of possible transmission of antibiotic resistant food borne bacteria to consumers and food handlers [48]. Similar multi-drug resistance phenotypes of *E. coli* isolated from retail chicken have been reported in studies conducted in Japan, Vietnam, Saudi Arabia and Slovakia [30, 31, 49, 50]. The common resistance phenotypes observed are of great clinical significance since these antimicrobials are considered to be among the frontline therapeutic drugs for treatment of most bacterial infections in humans in Kenya. Past studies assessing antimicrobial consumption in food producing animals in Kenya showed that tetracyclines, sulphonamides and trimethoprins, nitrofurans, aminoglycosides, beta lactams and the quinolones were the most commonly used drugs in food producing animals with tetracyclines and sulphonamides/trimethoprin topping the list in popularity and accounting for nearly 78% of the use [51]. A corresponding study in Tanzania showed similar results supporting these findings [52]. Such use has been shown to result in the development of resistant bacteria which can then reach heavily exposed individuals such as slaughter house workers, food handlers and farmers who feed the animals with the antimicrobials [53]. These resistant bacteria can then easily contaminate the carcasses of food animals along the production line to the retail outlets. In countries such as Uganda and Iceland, studies have shown that broiler chicken production has been associated with a high prevalence of antimicrobial resistance in isolates from these chickens due to consumption of antimicrobials [54, 55]. In this study, all samples from supermarkets, which had more resistant isolates, came from improved breeds of chicken/broilers while 80% of samples from low income areas were from local/indigenous chicken breeds. A common practice for broiler chicken producers in urban areas in Kenya is to add antibiotics into the commercial feeds or drinking water for chicken thus exposing them more to undue antimicrobial consumption [56]. This practice may modify the intestinal flora and create a selective pressure in favor of resistant bacteria [57]. On the other hand, indigenous chicken production in most African countries including Kenya is traditionally based on free range breeding systems allowing birds to grow without any external influence. They are estimated to reach a mature, marketable age by 8 months [42, 58].

Studies done in Burkina Faso, Korea and Lebanon also showed presence of *E. coli* contaminants harboring virulence genes in fresh poultry meat (with prevalence of 43%, 14% and 14% respectively) and that that ETEC, EPEC and STEC were the most common diarrheagenic *E. coli* detected [59–61]. This could be because ETEC, EPEC and STEC are much frequently implicated in various food and water borne diseases and they are known contaminants of meat and meat products [62, 63]. Studies, for instance in Canada, Spain and Minnesota U.S.A, have also shown that live chicken and other food animals are known reservoirs of these pathogenic *E. coli* and therefore contamination could actually be from the animal during evisceration or even from water used during their processing [64–66]. On the other hand EIEC and EaggEC are not implicated much in food and water borne illnesses and there are no known animal reservoirs for these pathogens hence any primary source of contamination appears to be infected humans [63, 67]. Another reason for this observation in this study is that ETEC had several virulent gene markers compared to the rest and thus could be easily detected more than the others. These results are important as they indicate that apart from being highly contaminated with coliforms which could lead to quick spoilage, raw retail chicken in Nairobi, Kenya is a potential source of food borne illnesses as it carries pathogenic *E. coli.* This has important implications and present unique challenges for interventions for microbial contamination of retail chicken meats in urban settings.
